# Holistic Health: Does It Really Include Mental Health?

**DOI:** 10.1100/tsw.2006.339

**Published:** 2006-03-14

**Authors:** Kimberly K. McClanahan, Marlene B. Huff, Hatim A. Omar

**Affiliations:** Division of Adolescent Medicine, Department of Pediatrics, University of Kentucky, Lexington, KY, USA

**Keywords:** holistic health, mind-body dualism, stigma, mental health parity, United States

## Abstract

Holistic health, incorporating mind and body as equally important and unified components of health, is a concept utilized in some health care arenas in the United States (U.S.) over the past 30 years. However, in the U.S., mental health is not seen as conceptually integral to physical health and, thus, holistic health cannot be realized until the historical concept of mind-body dualism, continuing stigma regarding mental illness, lack of mental health parity in insurance, and inaccurate public perceptions regarding mental illness are adequately addressed and resolved. Until then, mental and physical health will continue to be viewed as disparate entities rather than parts of a unified whole. We conclude that the U.S. currently does not generally incorporate the tenets of holistic health in its view of the mental and physical health of its citizens, and provide some suggestions for changing that viewpoint.

## INTRODUCTION

Although holistic health and nontraditional medicine have long been utilized in Eastern cultures, the areas of holistic health and medicine have only gained foothold in the U.S. in recent decades. At least two national organizations, the American Holistic Health Association (AHHA, founded in 1989)[1] and the American Holistic Medical Association (AHMA, founded in 1979)[2] promote holistic health and medicine as approaches to creating wellness by encouraging people to integrate physical, mental, emotional, and spiritual aspects of self and to take an active role in health care decisions and healing[1]. Holistic medicine uses both conventional and alternative therapies to prevent and treat disease and promote optimal health[2]. Alternative therapies/medicine are viewed as medicine outside the mainstream[3] (e.g., acupuncture, herbal healing, massage, naturopathy, therapeutic touch) and are often not covered by health insurance because of their nontraditional nature. Holistic health and medicine encompass not only the physical aspects (i.e., body) of a person, but also incorporate mental and emotional aspects (i.e., mind), inferring that physical and mental health are equally important and inseparable; therefore, there can be no health without mental health[4].

While viewing and respecting the "whole" person in the provision of health care seems a reasonable, even laudable notion, it appears that most health professionals in the U.S. do not actively or openly endorse the principals of holistic health, as surmised, at least in part, by the small membership of AHMA

(i.e., 1000)[2]. In fact, Davis-Floyd[5] defines the American medical system as "technocratic"[5] and defines twelve tenets of the technocratic model, the first of which is the separation of mind and body accompanied by the belief that the body is a machine, which, when it breaks down, is best fixed from an outside source. The body needs someone to manipulate and "fix" it rather than allowing that the body (in concert with the mind) has some ability to "repair" itself. When the body is viewed as a machine, it also allows the fixer to prescribe the correct remedy dispassionately without attending to a person's feelings, thoughts, or opinion in the matter. As Davis-Floyd states, the person becomes "the C-section in 112." Comparably, in the mental health field, the body and its behavior often become separated from the mind, and the illness becomes the entity without a person attached; hence, "the schizophrenic on Ward C," rather than a person suffering from schizophrenia. Additionally, in a technocratic model, authority and responsibility lie with the physician, not the patient, thus leaving the patient without choices because the physician is right. The technocratic model stands in sharp contrast to the holistic model; unfortunately, the technocratic model has long-standing philosophical roots that continue to be debated today.

## DESCARTES AND MIND-BODY DUALISM: A CONTINUING DEBATE

The separation of mind and body has long been a philosophical discussion in the Western world. Rene Descartes (1596–1650) is often noted as the philosopher who first fully explicated this separation by postulating the mind as the superior essence of humanity and spiritual essence, while the body was mechanical in nature and could be taken apart, studied, and repaired[5]; Descartes envisioned a mechanistic body where the mind pulled the strings[6]. Such thinking has contributed to a reductionistic view of health and mental health, and suggests that physical illness and mental illness are linear processes that come through purely biological processes[6]; additionally, the separation model completely ignores psychosocial and environmental factors, which research has increasingly shown to impact both physical and mental processes significantly[7,8,9].

Some researchers (i.e., neuroscientists) today discard Cartesian dualism by asserting that mental functions can be explained by brain science; other professionals (i.e., cognitive psychologists) assert that there are distinct psychological realms, but those realms are much like a computer, i.e., able to be measured and assessed[10]. However, this distinction still appears reductionistic and leaves out the context in which humans live and experience the world. It is difficult for the world to have meaning when human life is reduced to brain science and computer functions. Humans bring meaning to their worlds, constructing that meaning in the context of psychosocial, environmental, and cultural dimensions. Bracken and Thomas state, "Trying to grasp the meaningful reality of sadness, alienation, obsession, fear, and madness by looking at scans or analyzing biochemistry is like trying to understand a painting by looking at the canvas without reference to its wider world"[10]. Similarly, trying to look at physical illness or mental illness in a reductionistic manner is to lose the humanity and meaning, leaving out the context of a real person attempting to function in a real world. It is necessary to view the whole person, not just the symptoms or syndrome, whether those symptoms are assessed as primarily located in the body or the mind.

If one were able to define an illness as attributable to a pure biological etiology, it would be possible to view only the symptoms, to view the body as a machine to be worked on. Historically, with regard to mental illness, it was more difficult to look at symptoms and then prescribe the cure. However, with proliferating research suggesting that some mental illnesses have biological bases, it has become easier to do so. One need only take a pill and return for a follow-up in a month. As biological advances are made with regard to mental disorders, this has allowed the Cartesian notion to be applied to the mind in which the mind (i.e., brain) is reduced to a machine that does not work right, and a pill will fix it, if the right one can be found.

Part of the reason why psychiatry, psychology, and other mental health fields have been vilified as unscientific is because, historically, causes of mental illness have been elusive and less than quantifiable. The mind was not easily fixed. Since biological and psychopharmacological advances have been made, it appears that psychiatry has become more reductionistic through treating symptoms with medication. Interest in biological psychiatry has greatly increased while therapeutic interventions of a nonmedical nature have largely been left to those without medical degrees. Interestingly, even mental health professionals with nonmedical degrees are currently "under the gun" to use short-term manualized and evidence-based treatments. While interventions that work should be used, whether medication, manualized therapies, or clinical intuition and experience, it is less likely that a person can be a full partner in treatment for mental illness when the intervention is prescribed and the practitioner "knows" what is right based on that which is currently in vogue in the mental health and medical communities. There can also be no doubt that some of the drive to reduce treatment to a pill or a short-lived intervention based on what is written in a manual is a response to managed care insurance companies and the interventions for which such companies will and will not pay.

## INTERACTION OF MIND AND BODY AND THEIR EFFECTS ON EACH OTHER

Regardless of the continuing debate of mind-body dualism, many health and mental health professionals have come to understand, based on research and professional experience, that the mind and body interact with each other in complex ways and are a part of a unified whole person[7,9]. When something goes awry with the mind, the body may be affected by physical symptoms. The converse is also true. The two are inextricably intertwined and attention must be paid to both, as well as to the external environment.

There are several current theoretical models that incorporate a complex understanding of the mind and body in the context of the larger world. The biopsychosocial model is an approach to health purporting that the causes, development, and outcomes of an illness, whether physical or mental, are determined by the interaction of psychological, social, and cultural factors interfaced with biochemistry and physiology. The model asserts that physiology and biochemistry are not separate and distinct from the rest of life experience, and the mind and other body systems interact in ways that significantly impact health, illness, and well-being[9].

Another model, proposed and researched by Swedish psychologist David Magnusson, is a holistic-interactional model[11,12], an approach to human development that incorporates a blending of experience, mind, brain, and behavior, and incorporates reciprocal and holistic interactions that transpire among biological, psychological, and social-contextual processes[13].

From a health care perspective, holistic health care models provide a context in which the "mind and body are reunited"[5]. Holistic health care models incorporate the oneness of body-mind-spirit and suggest that the treatment of all states of illness should be conceptually integrated and treated in the context of the whole person encompassed by a whole life perspective. Mental and physical health should not just be viewed as equally important in terms of the resources and value that are placed on them, but as conceptually integrated and unified aspects within the whole person.

The psychological models enrich and enhance understanding regarding the manner in which humans develop over time and how a myriad of factors influence health or illness over the life span. Both models view the mind as an important component of human development and health, both mental and physical. The holistic health care model takes understanding a step further by viewing and treating body and mind as unified entities. Unfortunately, these models (and others like them) seem not to have been disseminated beyond an audience of those who express interest in such matters, and the resulting impact has not reached the broader public or those who make decisions regarding how a nation's health dollars will be spent (i.e., legislators).

## MENTAL HEALTH IN THE UNITED STATES AND THE WORLD: HOW MUCH PROGRESS HAS BEEN MADE?

In keeping with the Cartesian notion of attending to body rather than mind, the first U.S. Surgeon General's Report on Mental Health[7] was not published until 1999, although in the U.S., mental disorders account for more than 15% of the overall burden of disease from all causes and affect one in five Americans[7]. Major depression alone ranked only second to ischemic heart disease in magnitude of disease burden. Schizophrenia, bipolar disorder, obsessive-compulsive disorder, panic disorder, and post-traumatic stress disorder also contributed significantly to the burden represented by mental illness[7]. Clearly, a significant percentage of the population will suffer, at one time or another, from mental distress of some sort.

The Surgeon General's report rejects Cartesian dualism and asserts that mental health and illness are inseparable from physical health and illness, and defines mental health as "a state of successful performance of mental function, resulting in productive activities, fulfilling relationships with other people, and the ability to adapt to change and to cope with adversity"[7]. As early as 1948, the World Health Organization (WHO) defined health as the presence of well-being — physical, mental, and social — not as the absence of disease. How different are those definitions of health and mental health? Not very! In claiming that mental and physical health are inseparable, the Surgeon General's report called for many reforms in mental health care, not the least of which was mental health parity (i.e., equal reimbursement for physical and mental illness) with regard to health insurance. Seven years after the Surgeon General's report, parity has still not been achieved, and Medicare, the federal health insurance program after which many private insurance companies model their reimbursement practices, still pays substantially less for mental than physical illness. The WHO's 2005 Mental Health Atlas[14] states that the U.S. currently spends 13.9% of its total budget on health, with 6% being spent on mental health. The 1999 Surgeon General's Report[7] reported that 7% of the national health budget was spent on mental health. Thus, mental health dollars are dwindling, not increasing.

Not only is there a discrepancy between insurance reimbursement with regard to mental health, the U.S. remains sadly lacking with regard to any insurance coverage for its citizens; therefore, all aspects of health are often less than adequately addressed. It is estimated that 15.6% of Americans were uninsured in 2003 — an increase from 15.2% in 2002; 11.24% of children lacked health insurance in 2003[15]. In 2005, according to the WHO Atlas, the uninsured in the U.S. was approximately 16% (i.e., 47 million people), an additional increase since 2003. One reason for the increase in uninsured Americans is that employer-sponsored insurance coverage continues to erode due to high health care costs and employers' unwillingness to incur such costs for their employees[15]. When people do not have health insurance, health care needs often go unmet because health care services are unaffordable.

At the farthest end of the continuum are those who die from lack of health care. The Institute of Medicine (IOM) estimates that each year, 18,000 Americans between the ages of 25 and 64 die due to lack of insurance coverage[15]. It seems unconscionable that a wealthy, industrialized world leader, like the U.S., allows a substantial number of its citizens to live without health insurance. Many of those uninsured are not eligible for publicly funded health insurance because they work and may have incomes above the poverty line, but they cannot afford to pay for health insurance, and their employers do not offer it.

Worldwide, efforts have also been made to increase mental health knowledge, access, and funding[14]. Nevertheless, the results are dismal with regard to mental health resources, both in developing and developed countries. The 2005 WHO Atlas shows no increase in mental health resources since the first Atlas was published in 2001, although WHO estimates that 13% of all disease burden is caused by neuropsychiatric disorders[16]. Most countries spend only a small amount of their budgets on mental health; one-fifth of more than 100 countries supplying figures published in the 2005 Atlas spend less than 1% of their health budget on mental health.

Other worldwide initiatives also suggest that mental health is not seriously considered on a global front. The United Nations formulated Millennium Development Goals (MDG), to be achieved by 2015[4,17]. The MDG outlines goals in eight problem areas, three of them related directly to health: (1) the eradication of extreme poverty and hunger, (2) reduction of child mortality, and (3) improvement of maternal health[4]. Each of those three areas has been shown to be impacted directly by risk factors for mental illness[18,19,20], but no plans were made in the MDG to include interventions for mental health[4]. This lack of recognition of mental health risk factors and their impact on physical health again draws attention to the disparate thinking about body and mind.

## IMPEDIMENTS TO SEEKING MENTAL HEALTH CARE

There can be no doubt that there are many impediments to enhancing mental health and well-being. Practically, people may not seek mental health care because of lack of insurance or lack of insurance parity for mental health care. Philosophically, they may not seek such services because mental health is still seen as less important than physical health and is additionally seen as the domain of the weak and impaired, thus deeming mental health treatment as treatment sought only by those who cannot "pull themselves up by their bootstraps" (e.g., it isn't that bad, get over it, what's your problem). Physical illness, often still seen as only a biological problem, does not usually carry stigma. Mental illness, conversely, continues to do so[4,6,7,8,18], and that stigma stems, at least in part, from dualism where bodily complaints are legitimate and psychological/mental ones are not; thus, the historical and philosophical delineation of body and mind continues to impact mental health and wellness[7].

Other related variables also contribute to stigma regarding mental illness. Historically, in the U.S., people with mental illnesses were deemed "lunatics" and were primarily cared for by family. As the nation became more industrialized in the 19th century and families were less able to care for their own, mental health treatment was separated from general health treatment[7]; additionally, those with mental illness were often forced to undergo inhumane interventions now known to be extremely harmful.

Nationally, representative surveys have tracked public opinion regarding mental illness since the 1950s[21,22]. Early opinion surveys showed that the public viewed mental illness as a stigmatized condition[21,22]. More recent surveys[23] regarding public knowledge of mental illness have shown that the public is more informed about mental illness than in the past, but greater knowledge did not decrease social stigma[23], and the public currently believes that mentally ill people are more violent than the public at large[7,23]. While that belief is inaccurate, it does not facilitate the public's understanding of or compassion for the mentally ill. Furthermore, while the public is currently more willing to agree that people should be treated for mental illness, the public does not want to pay for such treatment with increased taxes or other public dollars[7].

Other research has shown that there is a label effect for the mentally ill; those with mental illness are more severely stigmatized than those with other illnesses[24]. Moreover, those with more severe mental illnesses are more severely stigmatized than those with less severe mental illnesses (e.g., psychotic disorders vs. depressive disorders)[25,26]. The impact of stigma is seen in the general health care system as well as with studies showing that people with mental illness receive fewer medical services than those not labeled as mentally ill. Additionally, those labeled with mental illness are less likely to receive the same range of insurance benefits as people without mental illness[24].

Public identification as "mentally ill" yields few benefits and may produce much harm; thus, it seems reasonable to avoid a label of mental illness. Research has suggested that people with concealable stigmas, such as mental illness, may choose to conceal that status (i.e., deal with it privately to the best of their ability) rather than risk the discrimination and labeling their status will likely confer on them[27]. Others avoid the stigma altogether by denying their group status (i.e., mentally ill) and by not seeking the help (i.e., mental health care) that would label them as a member of the group[24].

Given the foregoing, can it be any surprise that fewer than 30% of people with mental illness seek treatment for it[28] and that up to 60% of all visits to primary care physicians have a psychological basis[29]? Rather than seek the specialized mental health care they need, people go to their primary care physicians who, often as not, do not diagnose the relevant mental health condition[30], and if a mental health condition is diagnosed, patients often get prescriptions for antidepressants and nothing else. Such treatment cannot be presumed to be adequate, but it is likely less stigmatizing than seeking out the specialized care offered by mental health professionals.

## BRINGING BODY AND MIND TOGETHER

From a mental health perspective in the 21st century, Cartesian dualism, where the body (i.e., the machine that is an appendage) is attended to while the mind (i.e., the reported seat of reason and humanity) is not, seems untenable. It is akin to fixing the body of a car while ignoring the engine that makes it run. The link between psychological and physical health is clear[31], and there can be no health without mental health as a partner in the health care arena. In 2004, Russ Newman, Executive Director for Professional Practice of the American Psychological Association, in discussing the need for mental health parity in America, stated an ambitious goal when he said that someday the public might have “the recognition that good physical health is actually part of good psychological health”[31]. Unfortunately, that time seems far in the future, and while it appears there is greater recognition of the importance of mental health as a component of overall health in the U.S. at this time, it cannot be said that people are viewed as unified “wholes” where health care always considers all aspects of the person and mental health is viewed as one unique aspect. The whole person is not automatically considered when health care decisions are made.

In order to continue toward the goal of holistic health and holistic health care in the U.S., a number of efforts must be made. The belief in the separation of mind and body must continue to be challenged. As people are viewed more readily within the context of a holistic and unified personhood, greater strides can be made toward holistic health care. The public and those who make funding decisions regarding health care (i.e., legislators) must come to understand that physical and mental health are interactive and are gained (or lost) in the context of environmental factors, such as poverty, education, community, and social status.

Continuing efforts must be made to erase stigma as it relates to mental health and illness. If the public understands the complex interactions between mind and body in the context of environment, some stigma may be erased. Additionally, education regarding holistic health and the unity of the body-mind-spirit may also help to erase stigma by helping the public to understand that a person’s health and illness are interactive and a function of the differing aspects of a unified whole. Therefore, information from studies regarding these issues must be disseminated to the public in a manner that the public can understand and incorporate into its thinking. Accurate information must be disseminated about the symptoms of mental illness, and inaccurate beliefs (e.g., mentally ill people are more violent than others) must be challenged and discredited. Accurate information may help decrease negative labeling, discrimination, and negative attitudes against those with mental illness, allowing them to more readily seek appropriate treatment and participate in society as productive, valued citizens. Additionally, the public must be made aware of the high cost of untreated mental illness in society and must come to understand the potential savings, both in terms of dollars and human life, inherent in appropriate mental health care.

As a separate but related point, insurance parity for mental health must continue to be pursued and realized. The public as a whole will not view mental health as equally important to physical health as long as society at large discriminates financially against those with mental illness. The message of financial inequity with regard to health insurance is clear: Mental health is less important than physical health. That message must be changed. Only when equal amounts of money are allocated for mental and physical health will the public come to a better understanding that mental health is as important as physical health. The public, to some degree, believes what its leaders implement as policy; thus, if the public sees its leaders funding heath care for the whole person, public opinion regarding the importance of mental health will likely improve. When that occurs, it should also impact perceptions regarding those with mental illness and stigma should be lessened.

Education regarding mental health and wellness must also be more fully incorporated into training for medical providers, especially those training to practice in primary care and pediatric settings. Early detection of mental health problems can lead to early intervention and referral, thereby potentially alleviating or mitigating long-term cost-intensive care for mental illness.

Professional organizations and other entities promoting mental health and wellness need to increase public information campaigns and advocacy for mental health as a part of overall health. The American

Psychological Association's Mind/Body Health Campaign is one such public information effort currently in progress. Public information campaigns are also venues through which stigma can be addressed.

Finally, the language used within the health care arena must be changed. For example, when speaking of health, the word "health" is usually used, with "mental health" as a separate component; this continues to separate mind from body and does not allow for the concept of holistic health. Even those who practice holistic health care have difficulty not using the words "body" and "mind"[5], even though such practitioners may think of the "'bodymind' to indicate that it is all one thing." Our language needs to be used to incorporate all health, both of mind and body, and mental health providers should be designated as health care providers even if they are not medically trained. Such a change in the use of language will increase the chances that people will perceive health as an overarching aspect of personhood, with mental and physical health as equally important, but unique, aspects of a person, thereby increasing the probability that holistic health care in the U.S. will become a reality in the future.

## BIOSKETCHES

**Kimberly K. McClanahan, PhD,** is Assistant Professor of Pediatriacs at the University of Kentucky in Lexington. She has publications in adolescent substance use prevention and women with disabilities. E-mail: kimberly.mcclanahan@uky.edu

**Marlene B. Huff, PhD,** is an Associate Professor of Pediatrics at the Kentucky Clinic, University of Kentucky in Lexington. She has publications in the area of disability studies and various aspects of clinical social work. E-mail: marlene.huff@uky.edu

**Hatim A. Omar, MD,** is Professor of Pediatrics and Obstetrics/Gynecology and Director of Adolescent Medicine and Young Parent programs at the Kentucky Clinic University of Kentucky in Lexington. He has numerous publications in child health, adolescent health, medicine, obstetrics and gynecology. E-mail: Hatim.Omar@uky.edu
